# Fabrication of Multi-Walled Structure through Parametric Study of Bead Geometries of GMAW-Based WAAM Process of SS309L

**DOI:** 10.3390/ma16145147

**Published:** 2023-07-21

**Authors:** Jay Vora, Rudram Pandey, Pratik Dodiya, Vivek Patel, Sakshum Khanna, Vatsal Vaghasia, Rakesh Chaudhari

**Affiliations:** 1 Department of Mechanical Engineering, School of Technology, Pandit Deendayal Energy University, Raisan, Gandhinagar 382007, India; jay.vora@sot.pdpu.ac.in (J.V.);; 2 Journal of Visualized Experiments, Delhi 110016, India

**Keywords:** Gas Metal Arc Welding (GMAW), SS309LL, Wire-Arc Additive Manufacturing (WAAM), passing vehicle search (PVS) optimization, bead geometries, multi-walled structure

## Abstract

In the present study, an attempt is made to investigate and optimize the bead geometries of bead width (BW) and bead height (BH) of SS-309L using an SS316L substrate by employing a gas metal arc welding (GMAW)-based wire-arc additive manufacturing (WAAM) process. The Box–Behnken design approach was used to conduct the trials of single-layer depositions with input variables of travel speed (TS), voltage (V), and gas mixture ratio (GMR). The developed multi-variable regression models were tested for feasibility using ANOVA and residual plots. The data obtained indicated that V had the most significant impact on BW, followed by TS and GMR. For BH, TS had the most significant impact, followed by GMR and V. The results of single-response optimization using a passing vehicle search (PVS) algorithm showed a maximum BH of 9.48 mm and a minimum BW of 5.90 mm. To tackle the contradictory situation, a multi-objective PVS algorithm was employed, which produced non-dominated solutions. A multi-layered structure was successfully fabricated at the optimal parametric settings of TS at 20 mm/s, of voltage at 22 V, and of GMR at 3. For multi-layer structures, fusion among the layers was observed to be good, and they were found to be free from the disbonding of layers. This revealed the suitability of the PVS algorithm for generating suitable optimal WAAM variables. We consider the current work highly beneficial for users fabricating multi-layer structures.

## 1. Introduction

Additive manufacturing (AM) is now preferred as a widely accepted technique over traditional manufacturing methods because it produces near-net-shaped components quickly and efficiently using various materials [1,2]. Presenting opportunities while minimizing expenses and enhancing efficiency has become increasingly appealing to manufacturers seeking to optimize their processes [3]. As a result, AM has gained significant traction in recent years, with many companies adopting it on a large scale to gain a competitive edge in their respective industries [4]. The aerospace, automotive, and biomedical areas are becoming more interested in metal additive manufacturing [5]. Wire-arc additive manufacturing (WAAM) is a technique that can save time, costs, and material when making components compared to other AM methods [6,7]. Three key categories used to classify AM for heat sources are electron beams, laser beams, and electric arcs. Electron and laser beams require metal powder as feedstock material, which restricts their production capability [8,9]. Due to this reason, these two techniques have certain limitations for larger-scale productions [10]. However, using an electric arc as a heat source is mainly suitable for producing intricate and complex large-scale components at lower cost and reducing material waste due to the more significant deposition rate [11,12]. The electric arc method uses metal wire as feedstock material, which has reduced cost compared to metal powder as feedstock material for the same proportion [13]. The WAAM technique is founded on automated welding methods, for example, gas metal arc welding (GMAW), gas tungsten arc welding, and plasma arc welding. The fabrication of thin, multi-walled structures with reduced cost and easier material deposition at a higher rate in the GMAW-based WAAM method makes it more favorable over other techniques [14,15,16]. However, GMAW-based welding is a complex process that depends on several factors, such as current intensity, shielding gas type, voltage (V), gas flow rate, gas mixture ratio (GMR), travel speed (TS), wire feed speed (WFS), contact tip-to-work distance, and torch angle. This means that the process parameters that govern the quality of specimens must be carefully optimized, as the required parameters differ for different grades of materials. Controlling these variables for a suitable multi-layered structure is essential [17]. Two important characteristics of bead morphologies are bead width (BW) and bead height (BH). Optimal parametric settings of WAAM parameters improve bead morphologies for the fabrication of multi-walled structures [18]. In recent studies, passing vehicle search (PVS) algorithms have been successfully executed for multiple production systems [19,20,21,22].

Kumar et al. [23] studied the optimization of GMAW-based WAAM for multi-layer bead deposition on steels. They aimed to optimize the process parameters for improved dimensional accuracy and mechanical properties of the deposited beads. They utilized an RSM design for single-layer depositions using the input variables of TS, V, current, and gas flow rate. Their findings revealed that TS was a vital factor, with 52.29% influence on BW response and 43% on BH response. The desirability function was employed to achieve optimal parametric settings, and a multi-layered structure was then fabricated. Le et al. [24] created parts from 308L stainless steel using the WAAM technique. The authors used a mix of experimental design and optimization with the help of ANOVA. Their results showed that the mechanical properties of the part made from the optimized process parameters were excellent, showing the importance of optimization. Chaudhari et al. [25] studied the effect of WAAM process parameters on a bead’s geometry for single-layer deposition, such as BW or BH, with the variance of parameters such as TS, WFS, and V. They used a Box–Behnken design for singe-layer deposits and found that WFS was the most significant factor for both BW and BH, followed by V and TS. Their study revealed that a multi-layered structure was successfully fabricated at optimized parametric settings at a TS at 141 mm/min, a WFS at 5.50 m/min, and a voltage of 19 V. Natryan et al. [26] used the Taguchi technique to study the effects of TS, welding current, and filler diameter on the quality of welded joints. By applying an orthogonal array design and statistical analysis, they found the optimal mix of parameters that enhanced weld quality by minimizing defects and improving bead geometry and weld penetration. Vora et al. [27] fine-tuned the bead shape for GMAW-based WAAM. They applied a Box–Behnken design (BBD) to perform bead-on-plate tests. They used analysis of variance (ANOVA) to examine the regression equations and employed a teaching–learning-based optimization (TLBO) method to determine the best input parameters. A minimum BW of 4.73 mm and a maximum BH of 7.81 mm for single-layer depositions were obtained. A multi-layered structure was fabricated at simultaneously optimized parameters, and the structure was found to be free from the disbonding of layers. Kumar et al. [28] used a genetic algorithm to obtain the best process parameters for WAAM with near-net-shaped deposition. They discovered that the GA efficiently obtained process parameters that led to near-net-shaped deposition with fewer layers. Liberini et al. [29] aimed to select optimal process parameters for wire arc additive manufacturing using a multi-objective optimization approach to find the optimal values for bead width and height, porosity, and deposition rate. Their approach effectively found the most optimal values for the targeted properties, such as BW and BH, porosity, and deposition rate. Another study conducted by Wang et al. [30] employed a multi-wire indirect-arc-directed energy deposition method. Their obtained results showed significant impacts of WFS, current, and angle between the wires on the employed process. The results of the microstructures and mechanical properties showed favorable results for the used methodology as compared to the conventional one. Mishra et al. [31] used the optimization approach for simultaneous topology and deposition direction in WAAM using a mathematical model and a combination of GA- and gradient-based optimization techniques. This resulted in an improvement in part quality and a reduction in manufacturing time compared to conventional methods.

Stainless steel (SS) has increased its popularity in WAAM processing. SS is highly valued for its capacity to resist corrosion, rust, and staining [32]. Owing to its excellent strength, durability, and aesthetic appeal, it is primarily preferred for various applications [24]. It is also used in the medical and aerospace industries due to its biocompatibility and excellent ratio of strength-to-weight [33]. SS309L is a heat-resistant alloy commonly used in the chemical and petrochemical industries. SS309L has increased carbon presence compared to other steels, which gives it improved high-temperature strength [34]. The alloy is also resistant to suffixation and carburization, which makes it suitable for use in environments where these processes occur [35,36,37].

Based on past studied work, minimal work has been presented on the experimental investigation and optimization of process parameters for bead morphologies using the GMAW-based WAAM process for SS309L. Thus, the current study attempts an experimental investigation and optimization of the bead geometries of BW and BH of SS-309L using an SS316L substrate by employing a GMAW-based WAAM process. The optimized set of parameters is used to fabricate a multi-walled structure. TS, V, and GMR are elected as WAAM variables based on preliminary experimental trials, machine limits, and recently studied work. At the same time, BH and BW are taken as responses of bead morphology. A BBD is used to generate an experimental matrix for single-layer depositions, and the obtained results are analyzed through ANOVA, residual plots, and main effect plots. WAAM variables are optimized through a PVS algorithm using the empirical relations developed through the BBD. The multi-walled structure is then successfully fabricated at optimal parametric settings. We consider the current work with optimized parametric settings to be highly beneficial for users fabricating multi-layer structures.

## 2. Materials and Methods

### 2.1. Experimental Setup and Plan

In the present study, a metal wire of SS309L with a 1.2 mm diameter was used, and the bead on the plate was deposited on stainless-steel 316L-grade substrate plates using GMAW-based WAAM. Table 1 shows the chemical compositions of the filler wire and substrate, respectively.

**Figure 1:**
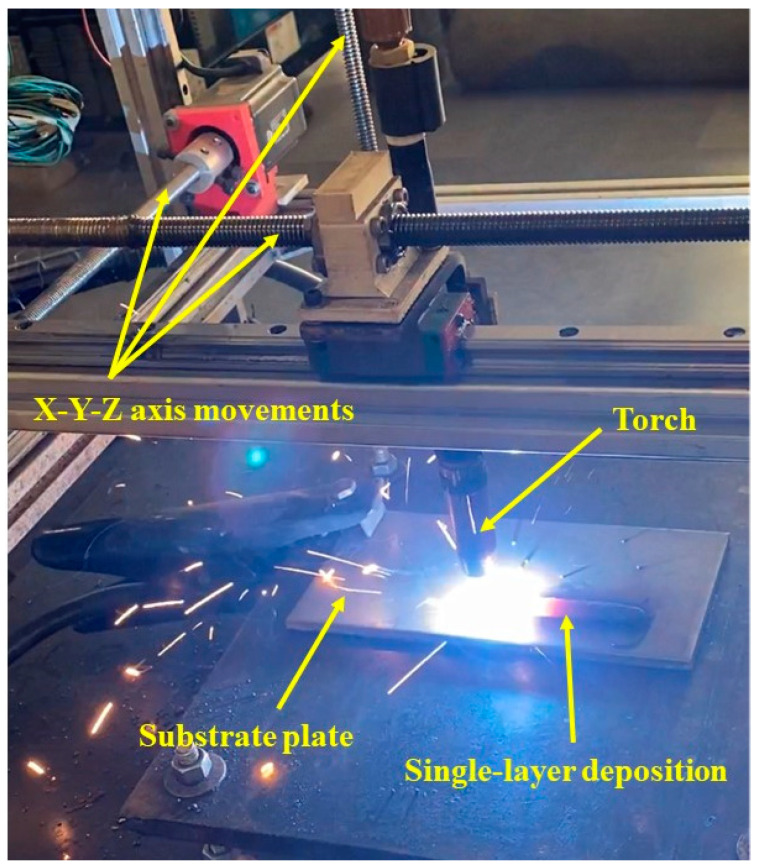
Experimental setup for GMAW-based WAAM process.

The GMAW process uses a temporary heat source to heat, melt, and solidify two parent metals and a filler material in a limited fusion zone to make a joint between the parent metals. An autonomous wire feeder constantly feeds the wire electrode via the tip of the torch, where the heat from the welding arc melts it. The distance between the end of the melted electrode and the molten weld pools and the transfer of molten metal to the weld pools controls the heat. The GMAW welding parameters determine the quality and cost of the welded joint. An ideal arc is formed if all the welding parameters are optimal and in accordance. Figure 1 displays the experimental setup used in the present study for the GMAW-based WAAM process.

The setup used in the study had the components of a wire feeder, a GMAW torch, shielding gas, a mixing chamber, and a controller. The build volume for the machine used was 220 × 220 × 500 mm. The torch was enabled to move in the x, y, and z axes to deposit the material on substrate plates. The controller was provided with input through G-code programming via a computer interface in the experimental setup. COLTON iFLEX 350 was used as a power source to heat and melt the metal wire.

A BBD was used to generate an experimental matrix for single-layer depositions on substrate plates using metal wire of SS309L. By using orthogonal arrays of a BBD, multiple factors can be tested with minimal experimental runs [38,39]. This approach helps systematically identify the essential factors that affect product or process quality [40]. A BBD also gives the relationships between a response variable and multiple input variables, such as polynomial regression models, to identify optimal input conditions that maximize the response variable [41,42]. TS, V, and GMR were elected as WAAM variables based on preliminary experimental trials, machine limits, and recently studied work. At the same time, BH and BW were taken as responses of bead morphology. GMR represented the proportions of CO_2_ gas, and the remainder was argon. Preliminary trials were carried out to identify the range of the selected variables following the BBD. Throughout the single-layer WAAM depositions, a weld bead length of 150 mm, an arc length of 3 mm, and a gas flow rate of 15 L/min were maintained. Table 2 displays the machining conditions used in the present study.

**Figure 2:**
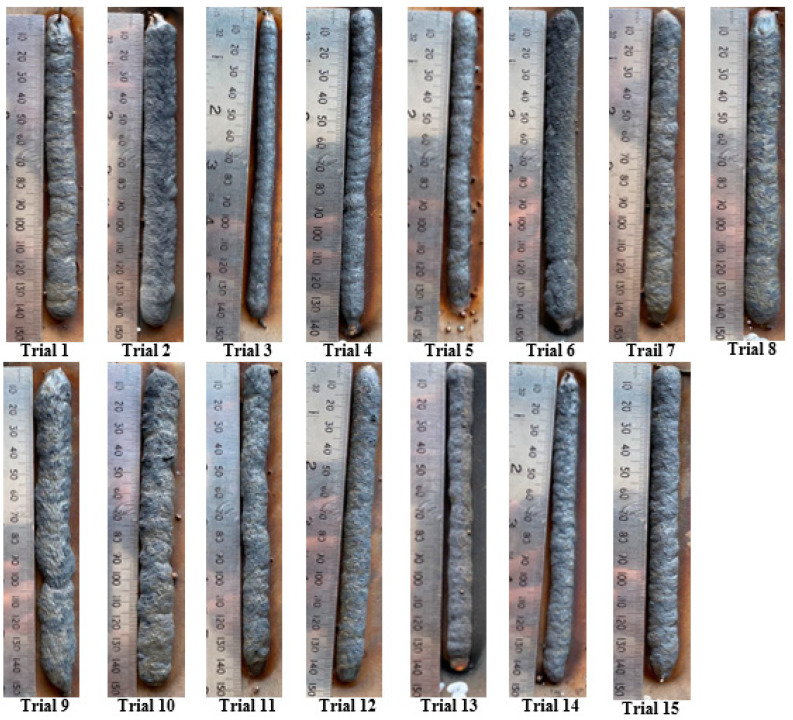
Single-layered deposition following the BBD.

The selected input parameters were varied at 3 levels, and 15 runs were carried out following the BBD matrix. The bead geometries of BH and BW were investigated for all the experimental trials. Figure 2 displays the single-layer depositions of fifteen trials by following the BBD matrix, as shown in Table 3 (run order). All the bead-on-plate samples were visually checked and found to be free of any lack of fusion, porosity, or any such defects. This established the workable parameter range of the selected study.

**Figure 3:**
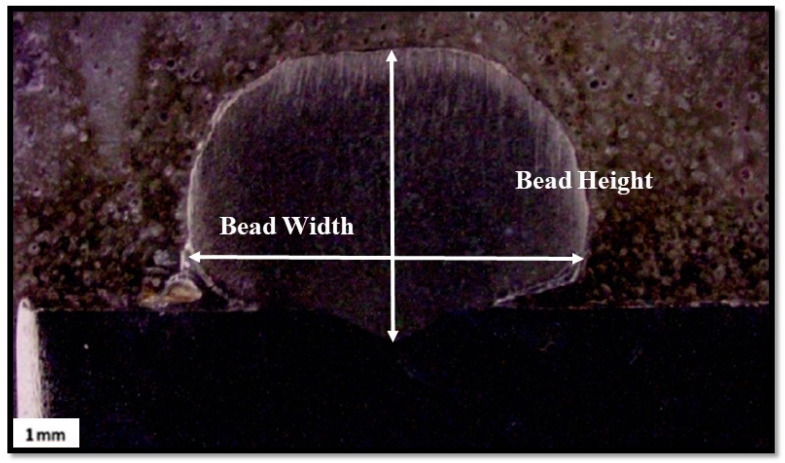
Procedure for determining bead geometries (BW and BH).

Optical microscopy was used to measure the bead morphologies of each single-layer deposition. Figure 3 represents the methodology used for the determination of bead geometries. Three different cross-sections of the bead depositions were cut to measure the bead geometries at various locations of the deposition. For better accuracy and more reliable results, their average values were considered in the present study.

### 2.2. Optimization Using PVS Algorithm

Savsani and Savsani [43] studied the passing vehicle search (PVS) algorithm, particularly for design problems related to engineering. The algorithm imitates the passing of a vehicle on a two-lane highway, emphasizing the principle of safe overtaking opportunities. The mechanism involves several interdependent and complex parameters, including the availability of gaps in oncoming traffic, the speed and acceleration of individual vehicles, road conditions, overall traffic, weather, and driver skill. While the PVS algorithm has demonstrated its usefulness, its application in real-world scenarios with various complexities and uncertainties still requires further investigation and refinement. The algorithm presented in this paper accounts for three types of vehicles (Oncoming Vehicle—OV; Front Vehicle—FV; and Back Vehicle—BV) on a two-lane highway. When a Back Vehicle desires to overtake a Front Vehicle, it must have a faster speed than the latter. Overtaking cannot occur if the Back Vehicle has a lower speed than the Front Vehicle. The algorithm also considers the speed and position of the Oncoming Vehicle and its respective distances and velocities when determining the feasibility of an overtaking maneuver.

On a two-lane road, there are three different vehicles (OV, FV, and BV) with different velocities (V_1_, V_2_, and V_3_), and their respective distances can be determined at any given time. The distance between BV and FV can be denoted as ‘x’, and that between FV and OV can be marked as ‘y’. Their velocities impose a primary constraint, where either FV’s velocity is slower than BV’s (V_1_ > V_3_) or BV’s velocity is slower than FV’s. If FV exceeds the speed of BV, overtaking becomes impossible, and BV maintains its desired velocity. It is possible to pass only if FV’s velocity is less than that of BV. An additional requirement for passing is that the distance between FV and BV during the maneuver is shorter than the distance covered by OV within the same timeframe. Thus, different conditions emerge for the selected vehicles. Employing a human-activity-based technique, the PVS algorithm models the passing behavior of vehicles and offers a meta-heuristic optimization approach capable of finding optimal or near-optimal solutions for given objective functions.

## 3. Results and Discussion

The BBD matrix of the RSM approach was utilized to obtain the results of BW and BH, as shown in Table 3. The most suitable condition for fabricating a multi-walled structure is to achieve maximum BH and minimum BW. The study also generated multi-variable non-linear regressions between the machining factors and the responses.

### 3.1. Empirical Relations for BH and BW

Multi-variable non-linear regressions were generated using Minitab v17 with the BBD of RSM to establish the relationships between the WAAM variables and bead geometries (BH and BW). These equations provided a starting point for evaluating response values beyond the experimental matrix of the BBD. Equations (1) and (2) are the regression equations for BH and BW, respectively, which were derived using the stepwise method of statistical approach with Minitab software.

(1) BH=36.81−0.356·V−1.613·TS−1.977·GMR+0.0258·TS·TS+0.0505 ·V·GMR+0.0337·TS·GMR

(2) BH=37.2−2.25·V−0.919·TS−0.724·GMR+0.0634·V·V+0.01683 ·TS·TS+0.02629·GMR·GMR+0.0255·GMR·GMR

### 3.2. ANOVA for BW and BH

A statistical method, analysis of variance (ANOVA), was used to determine the significance of the factors affecting the response variables. It measured the variability between different levels of the factors to determine which factors significantly impacted the response variables. The adequacy and reliability of the resulting regression equations were tested through an ANOVA analysis. Minitab v17 software was utilized to assess the significance of the model terms at a confidence level of 95%. Terms with probability values less than 0.05 were considered to significantly impact the response variables, while non-significant terms were treated as irrelevant [44].

Table 4 showcases the ANOVA results for BW and BH, and Table 5 shows the results of the model summaries for BW and BH. The statistical analysis of the output factors of BW and BH revealed significant contributions from the regression, linear, square, and two-way interaction models.

The three WAAM variables of V, TS, and GMR were all significant factors in the case of BW, while for the BH response, TS and GMR were substantial variables. TS had the highest contribution to BH, while V was observed to have the highest effect on BW. The small impact of the error term on all the responses indicated high accuracy in predicting values with minimal errors, and lack of fit was statistically non-significant, confirming the accuracy of the ANOVA results [45]. The model was deemed appropriate for predicting the output value as a result. The generated regression equations were deemed reliable and dependable for predicting the values of BW and BH, as evidenced by the significance of the model terms and a close-to-one R^2^ value, indicating effective prediction. The model’s effectiveness was evaluated by analyzing its R^2^ values. The R^2^ values obtained for BW and BH were 0.9921 and 0.9578. Such high R^2^ values suggest that the model’s predictions were accurate and closely matched the actual data. The model was a good fit for the data, as the R^2^ values were close to one.

### 3.3. Residual Plots for Output Measures

**Figure 4:**
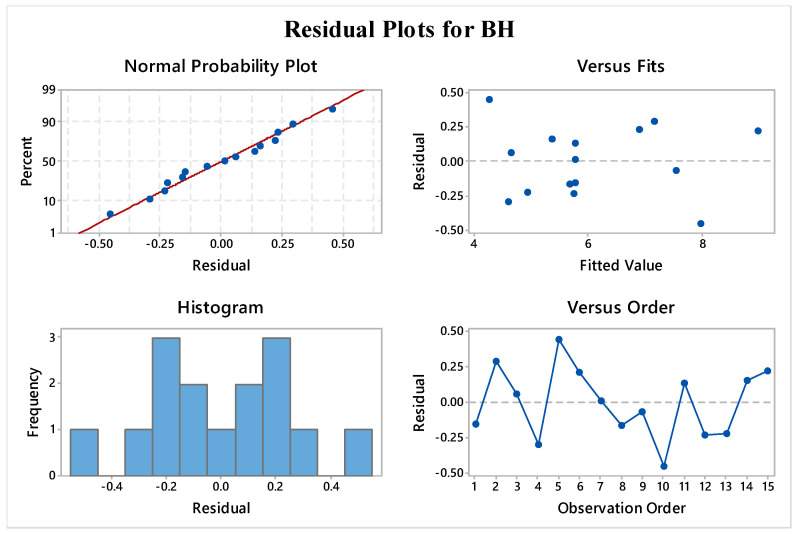
Residual plots for bead height.

To ensure the validity of the ANOVA model, specific assumptions must be met, and residual plots must be utilized to confirm the analysis outcomes [45]. Residual plots were used for the validation of the statistical results given by the ANOVA. The residual plots contained four individual analyses. Successful validation of these four plots confirmed the adequacy and reliability of ANOVA results and developed regression equations. Additionally, these validations meant that the regression equations could be used for the future prediction of outcomes for any parametric levels within the range of input parameters. The residual plots for BH, consisting of four plots, are presented in Figure 4.

**Figure 5:**
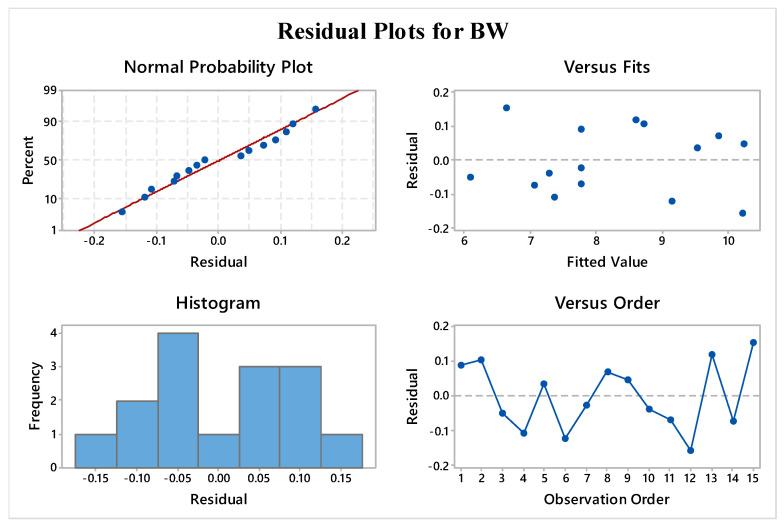
Residual plots for bead width.

The normality plot exhibited a linear trend that supported the ANOVA model’s suitability, indicating that the residuals followed a normal distribution. The versus fit plot also showed that the fits were randomly distributed around the source. In contrast, the histogram plot displayed a bell-shaped curve, which indicated the ANOVA data well. Furthermore, the absence of any particular pattern in the versus order plot further confirmed the ANOVA statistics, leading to better predictions of future outcomes. Figure 5 shows similar findings in the case of BW response. Thus, this shows that the generated regression equations were found to be adequate and reliable for future predictions of outcomes.

### 3.4. Main Effect Plots for Bead Width and Bead Height

**Figure 6:**
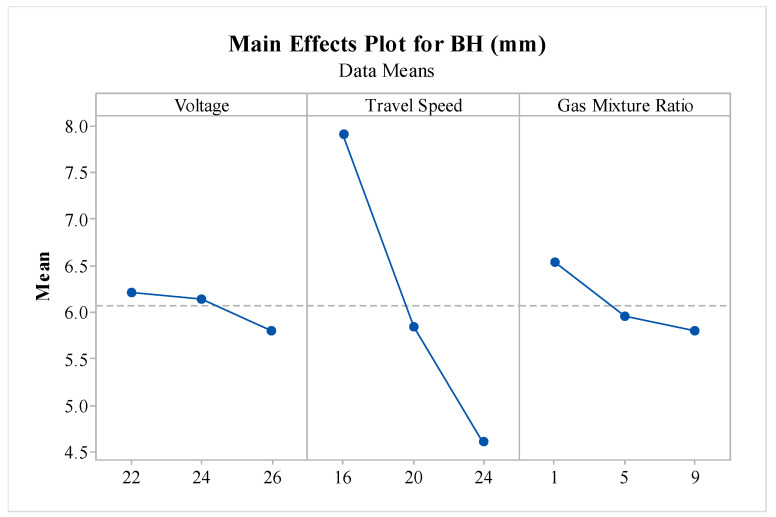
Effect of variations in WAAM variables on bead height.

The main effect plot, as shown in Figure 6, demonstrated the trends followed by V, TS, and GMR variance for bead height response, and it can be observed that a slight decrement in the BH of the deposited material was observed with an increase in V.

This phenomenon could be attributed to the increased heat generated through electrical resistance heating and the absorption of electrons onto the wire tip, providing the necessary energy for the melting and superheating of the wire electrode material [46]. The TS showed a trend wherein its increase led to decreased BH. This was because the higher movement speed of the torch allowed less time for deposition, leading to lesser BH [27,47]. The graph of BH vs. GMR shows that, with an increase in GMR value, the BH increased, and it also became obsolete when talking in relation to BH, as it just safeguarded the pool against air contamination. As per the main effect plots, the WAAM variables of V at 22 V, TS at 16 mm/s, and GMR at 1 were desirable to obtain the maximum BH value.

**Figure 7:**
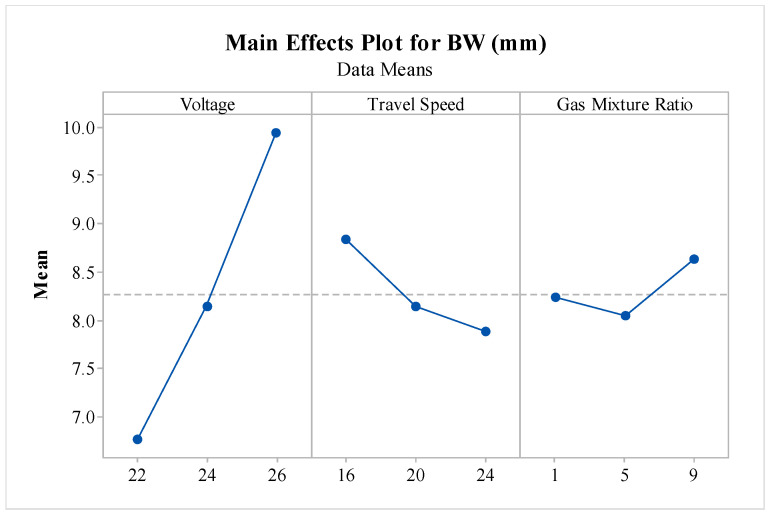
Effect of variations in WAAM variables on bead width.

The main effect plot, as shown in Figure 7, demonstrates the trends followed by V, TS, and GMR variance for bead height response. The plot of BW vs. V shows that the BW of deposition increased with an increase in voltage from 22 V to 26 V. This was due to higher voltage making wide arcs, leading to significant drops of molten metal [48]. The TS trend shows that the BW decreased with an increase in TS. This happened because, with higher TS, less metal could be dropped on the same position, leading to less deposition and, thus, smaller BW [25]. Finally, with higher values of GMR, the BW caused an insignificant change as, at first, it decreased and then increased, negating the changes or uncertainties caused. The gas mixture ratio, on the other hand, had minimal impact on the BW response. Its primary function was to protect the weld pool from atmospheric contamination, and it did not significantly affect the weld BW. As per the main effect plots, the WAAM variables of V at 22 V, TS at 24 mm/s, and GMR at 5 were desirable to obtain the minimum BW value.

### 3.5. Optimization of BH and BW Responses Using PVS Algorithm

The main effect plots of the response variables demonstrated the contradictory levels of WAAM variables for the optimal levels of BH and BW responses. This meant that the PVS algorithm was employed to optimize the responses. During the execution of the PVS technique, the upper and lower bounds of the design variables were considered as follows: 22 ≤ V ≤ 26; 1 ≤ GMR ≤ 9; and 16 ≤ TS ≤ 24. Table 6 shows the results of the single-response optimization.

Apart from voltage, the other two WAAM variables were observed at different levels. Additionally, the BW response value increased for the maximization of the BH response, which was not desirable, and vice versa. To validate the findings of the PVS algorithm, actual experiments were carried out. The smallest error between the results achieved from the PVS algorithm and validation trials resulted in good agreement between the bead geometry and the WAAM variables. This demonstrated the suitability of the PVS algorithm for the developed regression models.

Although the responses of BW and BH exhibited conflicting tendencies concerning the levels of WAAM design variables, it was crucial to determine the optimal design variable combination that could enhance both responses. This was necessary to achieve the desired quality in both BW and BH simultaneously. Owing to such reasons, multi-objective optimization was necessary. Table 7 shows the results of such an optimization, or Pareto points. Pareto fronts were employed to determine non-dominated solutions that satisfied the requirements of various industrial applications.

**Figure 8:**
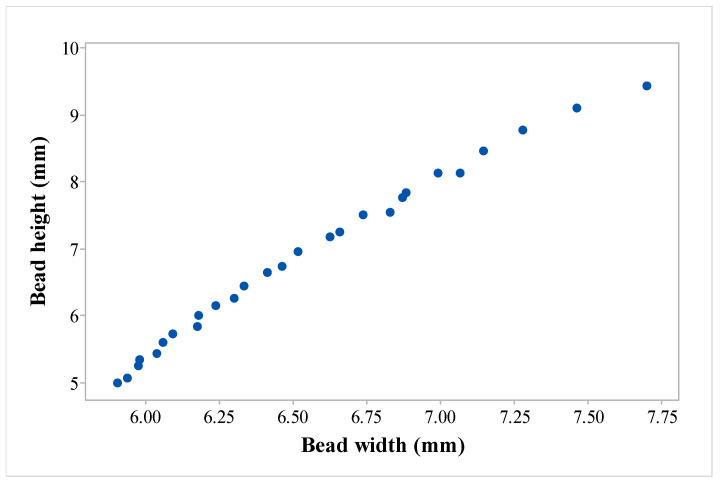
Pareto graph for BH vs. BW.

Figure 8 displays the Pareto graph of the BH vs. BW responses. Depending on the desired bead geometries needed for multi-layer thin-walled structure fabrication, a user can choose an optimal value from the available options.

### 3.6. Fabrication of Multi-Walled Structure

**Figure 9:**
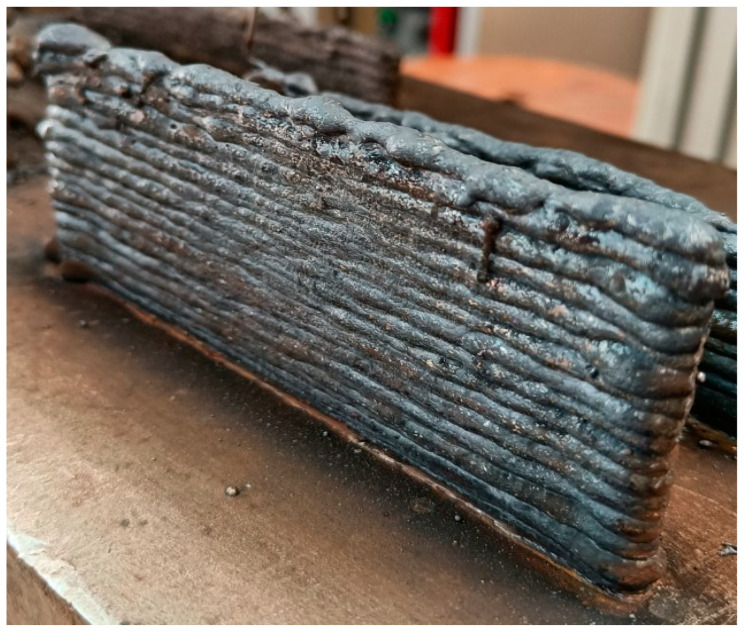
Multi-layer structure at optimal parametric settings.

An objective function was selected to fabricate a multi-layered structure by assigning equal importance to the BH and BW responses. The PVS algorithm was used for obtaining the levels of the WAAM variables, and it showed at the voltage of 22 V, the TS of 20 mm/s, and the DOP of 3 response values of BH at 6.46 mm and BW at 6.33 mm for single-layer deposition. A multi-layered structure was fabricated at these parametric settings. The multi-layer structure was fabricated at the optimal parameter settings of WAAM variables, as shown in Figure 9. For better geometry accuracy, the multi-layer structure was fabricated through layer-on-layer deposition following 180-degree turns of filler wire.

A cooling time of 60 s was applied between successive layers to reduce residual stresses. Fusion among the layers was observed to be good, and it was found to be free from the disbonding of layers. Some extra lumps of metal core were noticed on the extreme sides of the structure. However, these were effectively eliminated in post-processing. This revealed the suitability of the PVS algorithm for generating the suitable optimal WAAM variables. Therefore, the present work effectively demonstrated the requirement of having optimal parametric settings and the necessity of parametric optimization for fabricating thin, multi-walled structures using a GMWA-based WAAM process for SS-309L using an SS316L substrate. We believe that the present work may be useful for researchers and industrial applications to find optimal sets of parameters.

## 4. Conclusions

The present study used a GMAW-based-WAAM process for SS309L wire. V, TS, and GMR were identified as machining parameters, and BH and BW were output factors. Experiments were conducted following a Box–Behnken design. Optimization of bead geometries was obtained through the application of a PVS algorithm. Based on the key findings and results, the following conclusions can be drawn:

- Multi-variable non-linear regressions were generated among the WAAM variables and output responses.
- ANOVA was employed to validate the appropriateness and reliability of the obtained regression equations. The ANOVA revealed that the quadratic model, including linear, squared, and interaction model terms, was statistically significant for both the bead height and width responses. The lack of fit results signified the model’s suitability and acceptability for both responses. The validation results from the ANOVA of an R-squared value close to one showed that the model was adequate and acceptable.
- TS had the largest impact on BH response, followed by GMR, while TS followed V and GMR showed a substantial impact on BW response.
- The influences of the WAAM variables (TS, V, and GMR) were studied on the BH and BW responses. It showed conflicting situations for attaining the desired levels of bead geometries.
- Single-response optimization using the PVS technique obtained a maximum BH and a minimum BW of 9.48 mm and 5.90 mm, respectively. Pareto fronts were employed to determine non-dominated solutions that satisfied the requirements of various industrial applications.
- The multi-layered structure was successfully fabricated at optimal parametric settings of V at 22 V, TS at 20 mm/s, and DOP at 3. For the multi-layer structure, fusion among the layers was observed to be good, and it was found to be free from the disbonding of layers. This revealed the suitability of PVS for generating suitable, optimal WAAM variables.
- The present work effectively demonstrated the requirement of having optimal parametric settings and the necessity of parametric optimization for the fabrication of thin, multi-walled structures using a GMWA-based WAAM process for SS-309L using an SS316L substrate. We believe that the present work may be useful to researchers’ industrial applications to find optimal sets of parameters. As a result, in future work, fabricated multi-layered structures will be examined using microstructure investigations and mechanical properties such as tensile, impact, and microhardness testing.

## Figures and Tables

**Table 1:** Chemical compositions of substrate and metallic wire (SS316L).

Element	Cr	Ni	Mo	Mn	Si	C	P	S	N	Fe
**Metallic wire (SS309L)**	22–25	12–15	-	2	1	0.2	0.045	0.03	-	Balance
**Substrate plate (SS316L)**	17.09	10.61	2.38	1.17	0.59	0.013	0.011	0.011	0.09	Balance

**Table 2:** Experimental conditions of GMAW-based WAAM process.

Input Factors	Values/Levels
Travel speed, TS (mm/s)	16; 20; 24
Voltage, V (V)	22; 24; 26
Gas mixture ratio, GMR	1; 5; 9
Gas flow rate (L/min)	15
Weld bead length (mm)	150
Arc length, (mm)	3

**Table 3:** Results of bead geometries following the BBD.

Std. Order	Run Order	V (V)	TS (mm/min)	GMR	BH (mm)	BW (mm)
14	1	24	20	5	5.61	7.850
11	2	24	16	9	7.46	8.820
3	3	22	24	5	4.71	6.050
10	4	24	24	1	4.31	7.259
4	5	26	24	5	4.71	9.554
9	6	24	16	1	9.20	9.010
15	7	24	20	5	5.78	7.735
6	8	26	20	1	5.51	9.910
2	9	26	16	5	7.47	10.270
1	10	22	16	5	7.52	7.250
13	11	24	20	5	5.90	7.690
8	12	26	20	9	5.51	10.050
12	13	24	24	9	4.73	8.704
7	14	22	20	9	5.51	6.987
5	15	22	20	1	7.13	6.783

**Table 4:** ANOVA for BW and BH.

Source	DF	SS	MS	F	P
**Bead Width**					
**Regression**	7	24.0000	3.4286	125.53	0.000
**Linear**	3	22.3142	7.4381	272.33	0.000
V	1	20.2057	20.2057	739.39	0.000
TS	1	1.7889	1.7889	65.50	0.000
GMR	1	0.3169	0.3196	11.70	0.011
**Square**	3	1.0175	0.3392	12.42	0.003
V × V	1	0.2372	0.2372	8.68	0.021
TS × TS	1	0.2676	0.2676	9.80	0.017
GMR × GMR	1	0.6535	0.6535	23.92	0.002
**Two-way Interaction**	1	0.6683	0.6683	24.47	0.002
TS × GMR	1	0.6683	0.6683	24.47	0.002
Error	7	0.1912	0.0273		
**Lack of Fit**	5	0.1776	0.0355	5.22	0.169
Pure Error	2	0.0136	0.0068		
**Total**	14	24.1912			
**Bead Height**					
**Regression**	6	34.6626	5.7771	30.27	0.000
**Linear**	3	31.3517	10.4506	54.75	0.000
V	1	0.4689	0.4689	2.46	0.156
TS	1	29.4147	29.4174	154.13	0.000
GMR	1	1.4654	1.4654	7.68	0.024
**Square**	1	0.8587	0.8587	4.50	0.067
TS × TS	1	0.8587	0.8587	4.50	0.067
**Two-way Interaction**	2	2.4521	1.2261	6.42	0.022
V × GMR	1	0.8806	0.8806	4.61	0.064
TS × GMR	1	1.5715	1.5715	8.23	0.021
Error	8	1.5269	0.1909		
**Lack of Fit**	6	1.4711	0.2452	8.79	0.106
Pure Error	2	0.0558	0.0279		
**Total**	14	26.7947			

**Table 5:** Model summaries for BW and BH.

**Model Summary for BW**			
**S**	**R-sq.**	**R-sq. (adj.)**	**R-sq. (pred.)**
0.165266	99.21%	98.42%	94.36%
**Model Summary for BH**			
**S**	**R-sq.**	**R-sq. (adj.)**	**R-sq. (pred.)**
0.436877	95.78%	92.62%	89.73%

**Table 6:** HTS results for individual response geometries.

Conditions	Input Factors			Predicted Responses through PVS		Experimental Results	
	V	TS	GMR	BH	BW	BH	BW
Maximization of BH	22	16	1	9.48	7.70	9.69	7.59
Minimization of BW	22	24	2	5.01	5.90	5.17	6.02

**Table 7:** Pareto optimal points.

Sr. No.	V	TS	GMR	BH	BW
1	22	16	1	9.45	7.70
2	22	24	2	5.01	5.90
3	22	16	2	9.12	7.46
4	22	16	3	8.79	7.28
5	22	16	4	8.47	7.15
6	22	16	5	8.14	7.07
7	22	19	3	6.97	6.52
8	22	18	3	7.53	6.74
9	22	17	3	8.13	6.99
10	22	17	5	7.55	6.83
11	22	24	1	5.07	5.94
12	22	20	3	6.46	6.33
13	22	19	3	6.97	6.52
14	22	20	4	6.27	6.30
15	22	19	4	6.74	6.46
16	22	23	2	5.35	5.98
17	22	19	2	7.19	6.63
18	22	18	4	7.27	6.66
19	22	21	4	5.85	6.18
20	22	22	2	5.73	6.09
21	22	17	4	7.84	6.89
22	22	21	2	6.17	6.24
23	22	17	4	7.84	6.89
24	22	23	1	5.44	6.04
25	22	20	2	6.65	6.41
26	22	19	2	7.19	6.63
27	22	21	3	6.01	6.18
28	22	20	2	6.65	6.41
29	22	18	4	7.27	6.66
30	22	17	3	8.13	6.99
31	22	18	2	7.78	6.87
32	22	20	3	6.46	6.33
33	22	22	3	5.61	6.06
34	22	21	2	6.17	6.24
35	22	19	4	6.74	6.46
36	22	18	3	7.53	6.74
37	22	16	5	8.14	7.07
38	22	23	3	5.25	5.98
39	22	22	2	5.73	6.09
40	22	20	4	6.27	6.30
41	22	22	3	5.61	6.06
42	22	21	3	6.01	6.18
43	22	22	1	5.85	6.18
44	22	18	2	7.78	6.87

## Data Availability

Data presented in this study are available in this article.
